# The Ideal Time to Administer Pre-operative Antibiotics: Current and Future Practices

**DOI:** 10.7759/cureus.24979

**Published:** 2022-05-13

**Authors:** Dania Baseel, Juliana Kim, Sumayya Mohammed, Andrew Lowe, Javed Siddiqi

**Affiliations:** 1 Medical School, Boston University, Boston, USA; 2 Medical School, University of California Berkeley, Berkeley, USA; 3 Medical School, Arrowhead Regional Medical Center, Colton, USA; 4 Pharmacy, Arrowhead Regional Medical Center, Colton, USA; 5 Neurological Surgery, Riverside University Health System Medical Center, Moreno Valley, USA

**Keywords:** surgery, antibiotic prophylaxis, clindamycin, cefazolin, preoperative antimicrobials

## Abstract

Background

Preoperative antibiotic prophylaxis is a method of administering antibiotics prior to surgical procedures to decrease surgical site infections. The Center for Disease Control and Prevention (CDC) guidelines recommend administering the chosen antibiotic within 60 minutes prior to incision. However, further research can be conducted to explore and determine a more precise and ideal time for preoperative antibiotic prophylaxis.

Methods

This paper explores the most used antibiotics within the Department of Neurosurgery at Arrowhead Regional Medical Center, which are cefazolin and clindamycin, and pinpoints the ideal time of preoperative antibiotic prophylaxis based on peak serum levels. It will present and discuss findings by analyzing the pharmacokinetic profiles of each antibiotic, focusing on the minimal inhibitory concentration (MIC), time to peak in the tissue, and duration of action to determine the appropriate time for redosing.

Results

Our findings indicate that based on the pharmacological profiles, the ideal time to administer preoperative antibiotics for cefazolin is 40 minutes prior to incision, and for clindamycin is 45 minutes prior to incision.

Conclusions

This study may help guide clinical decision-making and lead to minimizing the rate of infections and decreasing hospital stay.

## Introduction

Surgical site infections (SSIs) have been the norm since the origins of surgery until the nineteenth century when the concept of antisepsis was developed [1]. The first statistics based upon operative mortality were published in 1841, by a French surgeon Joseph-François Malgaigne. Malgaigne reported that the average mortality of amputations was 60%, primarily caused by nosocomial infections [2]. Lord Lister was one of the first surgeons to suggest that anything in contact with wounds, including surgical sites, should be free of germs [1]. This application of germ theory to surgery had significant implications in the sterile practices used today. However, SSIs remain a significant operative complication. They occur in up to 2% of all surgical procedures and constitute over 20% of nosocomial infections [1]. These infections can prolong the length of hospital stay and inevitably increase total treatment costs. One study demonstrated that SSI increased total cost by over $20,000 and extended hospitalizations by 9 days [1].

Currently, the use of preoperative antibiotic prophylaxis has significantly decreased the incidence of surgical site infections. The Center for Disease Control and Prevention released guidelines for preventing SSIs in 2017 which discussed the use of parenteral antimicrobial prophylaxis prior to incision. The current timing guidelines for antibiotic administration are defined as within 60 minutes prior to incision. However, these guidelines fail to provide further details on the timing of antibiotic administration given the duration of therapy for the selected prophylactic antibiotic [3]. While it is widely accepted that the use of antibiotic prophylaxis preoperatively decreases the frequency of SSIs, the best time to administer the antibiotics is still under debate. One systematic review and meta-analysis covering over 54,000 patients found that the risk of SSI almost doubled when antibiotics were administered after the first incision compared to over two hours prior to incision but found no significant difference in the rate of infections when antibiotics were administered within one hour or two hours prior to incision [4].

The parameters that are necessary to accurately assess antibiotics for the best window of application to minimize post-surgical infections include tissue half-life, minimal inhibitory concentration, and redosing interval. Half-life is the amount of time required for the plasma concentration of the drug to reduce by half. This paper is focused on tissue concentration over serum concentration as tissue concentration is more specific for surgical infections. The minimal inhibitory concentration (MIC) is the concentration of antibiotics at the site of infection that inhibits bacterial growth. The MIC90 is the MIC at which 90% or more of bacteria are inhibited. Furthermore, dosing interval is important to maintain a bactericidal level of antibiotics in the system and has been shown to be neglected. The dose interval is 1-2 times the half-life of the drug and is measured from the time of administration rather than the start of the procedure.

The aim of our study is to investigate practices that would allow for the ideal timing of preoperative antibiotic prophylaxis. It is a quality metric that is nationally mandated and reported to the public by Medicare and Medicaid. Within our home department, the Department of Neurosurgery at Arrowhead Regional Medical Center (ARMC), the current policy is to follow the preoperative guidelines outlined by the CDC: 60 minutes prior to incision. ARMC practice reflects a dosing interval of 4 hours subsequently for both cefazolin and clindamycin. We believe that the ideal timing for administration of preoperative antibiotics will vary based on the pharmacological properties of each antibiotic and that it would be beneficial to begin surgical incision during the peak serum level specific to each antibiotic.

Mechanism of action and pharmacokinetics of two antibiotic agents

Cefazolin, a beta-lactam antibiotic, is a widely used first-line prophylactic agent for surgical procedures. It is the only first-generation cephalosporin in the United States and works by binding to penicillin-binding proteins, which play an important role in bacterial cell wall construction [5]. By inhibiting cell wall formation, cefazolin causes bacterial lysis [5]. It works well against gram-positive cocci, *Proteus*, *Escherichia coli*, *Klebsiella*, and *Staphylococcus​​​​​​​ aureus *[5]. Cefazolin is widely used for increased protection from bacterial infections: septicemia and respiratory, biliary, or genitourinary (GU) tract [6]. Cefazolin is also a widely used alternative in patients that have a penicillin allergy [5]. It has a large volume of distribution except for poor penetration of the blood-brain barrier, and it is mostly excreted unmodified through the kidneys [5,7].

Clindamycin is also primarily excreted through urine [8]. It is classified as a lincomycin antibiotic, frequently used for over 50 years as a prophylactic treatment for surgery and the cure of other bacterial infections [8]. Clindamycin works by slowing or stopping the rate of growth of bacteria through the interruption of the transpeptidase reaction. This agent reversibly binds to ribosomal subunits, inhibiting chain elongation and preventing peptide bond formation [9]. Additionally, it alters the bacterial cell wall, inhibiting the adherence of bacteria to host cells [10]. Clindamycin is indicated in the treatment of serious infections due to certain susceptible strains of *Streptococci*, *Pneumococci*, and *Staphylococci*. Clindamycin is dispersed throughout the body fluid and tissues, with the exception of non-significant levels in the cerebrospinal fluid (CSF). 

## Materials and methods

**Figure 1:**
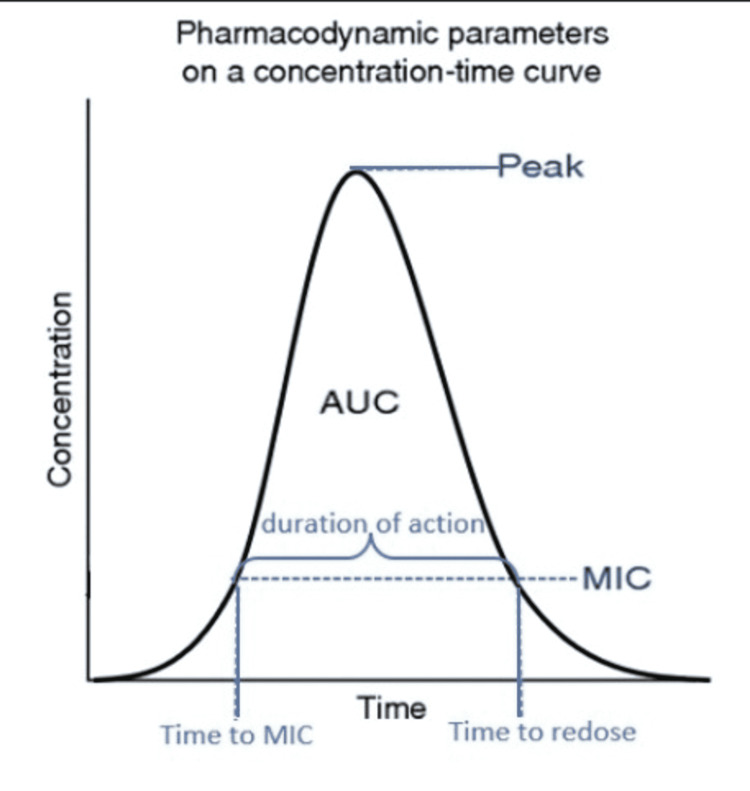
This figure depicts how to read pharmacodynamic parameters on a graph. MIC: minimal inhibitory concentration; AUC: area under the curve

To analyze our proposal, we chose the most commonly used antibiotics within our home department, the Department of Neurosurgery at Arrowhead Regional Medical Center which were cefazolin and clindamycin. Our team obtained data from existing literature on cefazolin and clindamycin pharmacodynamics when administered intravenously in adults with normal renal function. The following factors were deemed relevant: serum half-life, time to peak serum concentration, minimal inhibitory concentration (MIC), time to peak in tissue, and dosing interval. All of these are parameters of time except for the minimal inhibitory concentration which is an important value because it represents the smallest concentration of antibiotic needed in tissue to remain bactericidal. It is dependent upon the bacterium, the affected (in vivo) human, and the antibiotic utilized to combat the bacterium [11]. The aforementioned factors allowed us to analyze the pharmacokinetic profile of each antibiotic to determine the ideal timing of preoperative administration. Figure 1 represents how the pharmacodynamic parameters of the chosen antibiotics will be depicted.

## Results

**Figure 2:**
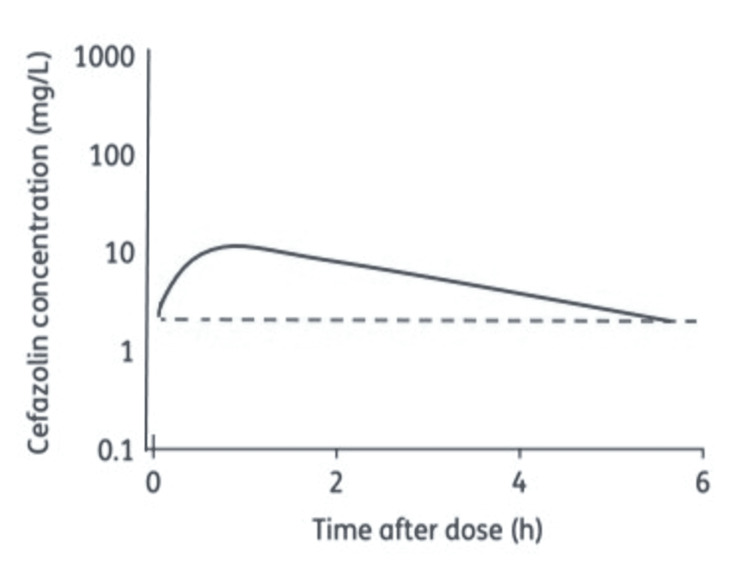
Cefazolin concentration over time. This figure is modified from Roberts et al. [16]. It depicts the observed tissue concentration of cefazolin after dosage in critically ill post-trauma patients. The dashed line is representative of the MIC90 of cefazolin.

**Figure 3:**
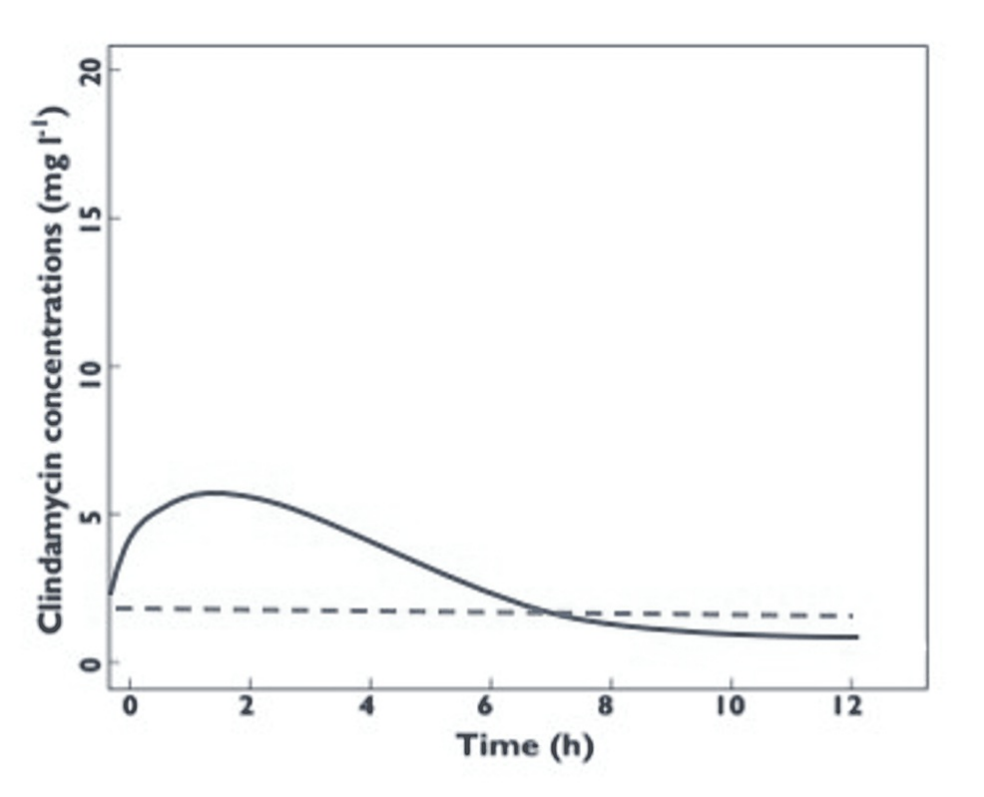
Clindamycin concentration over time. Modified from Bouazza et al. [18]. This graph depicts the observed clindamycin concentrations and population pharmacokinetic model-predicted clindamycin concentrations (curve) as a function of time for intravenous infusions. The dashed line is representative of the MIC90 of clindamycin.

Table 1 organizes data on the pharmacokinetic parameters of cefazolin and clindamycin. This information is necessary to analyze the best time for preoperative administration of the antibiotics. Current literature was reviewed to create the following Figures 2, 3 which depict the natural course of cefazolin and clindamycin (respectively) in tissue. 

**Table 1:** Pharmacokinetic parameters for cefazolin and clindamycin after intravenous administration. [5, 12, 13, 14, 15, 16, 17]

	Serum Half-Life	Time of Peak, serum	Minimal Inhibitory Concentration (MIC_90_)	Duration of Action	Redosing Interval
Cefazolin	2 hours ^5^	40 min ^15^	3.5 mcg/mL^16^	~2-5 hours ^13^	2-4 hours^17^
Clindamycin	2.4 hours^12^	45 min ^13^	2 mcg/mL^14^	12+ hours ^13^	3-6 hours ^17^

## Discussion

Cefazolin has a narrower time frame for incision, as well as a shorter redosing interval (Table 1). According to the graph, cefazolin has a MIC of 3.5 mcg/mL and reaches peak concentration at 40 minutes within intravenous administration, then immediately starts to decrease as time passes (Figure 2) [15]. The figure demonstrates that the most effective time for incision is 40 minutes post-administration and that the antibiotic remains productive for 4 hours. Both figures are aligned with standard preoperative doses of each antibiotic, 2 g of cefazolin and 1 g of clindamycin. As seen in Figure 3, the tissue concentration of clindamycin reaches above the MIC90 at about 45 minutes post-administration and remains detectable for almost 6 hours into the procedure [19]. Therefore, clindamycin proves to be an appropriate preoperative prophylaxis, contributing ample tissue concentrations for 3-6 hours until another dose is required [17]. Subsequently, our recommendation is that cefazolin should be administered 40 minutes before incision and clindamycin should be administered 45 minutes before incision to ensure that the antibiotics will be at their peak concentration during the procedure. However, we recognize that a 15-minute delay in either direction may provide results that are just as effective if not more so depending on the surgery. Additionally, the antibiotics should be redosed every 2 or 3 hours respectively for cefazolin and clindamycin to deter any chance of the antibiotics falling below MIC90 at any point during the operation.

Cefazolin and clindamycin differ in many ways, including their metabolism. Clindamycin is metabolized by the liver whereas cefazolin is cleared through the kidneys and has been known to cause acute interstitial nephritis in some cases [20,21]. Abnormal renal function could lead to an imbalance and failure to excrete the dose of antibiotic given. Cefazolin levels were shown to be markedly persistent in uremic patients [21]. The change in pharmacokinetics would skew dosing so we chose to focus on adults with normal renal function. Clindamycin is used instead of cefazolin to treat susceptible organisms in renally impaired patients and those that are allergic to beta-lactams.

Along with clindamycin and cefazolin, another commonly utilized antibiotic is vancomycin. However, we decided not to include vancomycin in our proposal because the dosing is heavily based on many factors including renal function, body weight, age, and gender. Vancomycin exists as a powerful antibiotic against gram-positive bacteria that should be administered slowly and monitored closely to avoid as best as possible nephrotoxicity and resistant *S. aureus* [22]. It has a rapid onset of action, peaking almost immediately after intravenous administration, but unlike cefazolin and clindamycin, there is no baseline time frame of incision or redosing interval in any previous studies or research. Therefore, we are not in a position to refine the ideal time to administer this preoperative antibiotic.

Possible future studies can feature both retrospective and prospective reviews. In terms of the retrospective studies, infection rates after surgical procedures can be compared to the timing of antibiotic administration. Additionally, data can be collected to observe the compared peak time of pediatric and adult patients without normal renal function. A statistical power ratio can be conducted to determine the necessity of administering preoperative antibiotics at ideal timing, maximizing their therapeutic output. As for prospective studies, it may be beneficial to explore the significance of preoperative antibiotic administration based on the ideal timing defined in our study. Additionally, a larger sample size would be necessary in order to implement a power ratio on the data for a more nuanced picture.

## Conclusions

We sought to decrease the rate of SSIs in all surgical procedures by utilizing the most used antibiotics within the Department of Neurosurgery at Arrowhead Regional Medical Center. Our findings indicate that the ideal time to administer preoperative antibiotics stands at 40 minutes prior to the incision for cefazolin and 45 minutes before incision for clindamycin because these antibiotics will be at peak levels in tissue. Furthermore, both antibiotics should be readministered during the earlier end of the redosing range to maintain a high tissue concentration. This information provides the basis for further research in the field of post-surgical infections, as we can help elucidate the true ideal time for antibiotic prophylaxis by further testing.
